# Impact of the Finnish Maternity Grant on infant mortality rates in the 20th century: a natural experimental study

**DOI:** 10.1136/jech-2022-219488

**Published:** 2022-10-27

**Authors:** Ronan McCabe, Srinivasa Vittal Katikireddi, Ruth Dundas, Mika Gissler, Peter Craig

**Affiliations:** 1 MRC/CSO Social & Public Health Sciences Unit, University of Glasgow, Glasgow, UK; 2 Department of Knowledge Brokers, THL, Helsinki, Finland; 3 Research Centre for Child Psychiatry and Invest Research Flagship, University of Turku, Turku, Varsinais-Suomi, Finland; 4 Academic Primary Health Care Centre, Region Stockholm, Stockholm, Sweden; 5 Department of Molecular Medicine and Surgery, Karolinska Institutet, Stockholm, Sweden

**Keywords:** INFANT MORTALITY, INFANT, NEWBORN, HEALTH POLICY, CHILD HEALTH

## Abstract

**Background:**

Baby boxes provide goods to new parents and a space for infant sleeping. They were first introduced in Finland, and it has been argued that the policy helped reduce infant mortality. We evaluated the impact of the Finnish Maternity Grant (which includes the Finnish Baby Box) on infant mortality rates (IMRs) at the points of introduction (disadvantaged mothers only) in 1938 and universalisation in 1949.

**Methods:**

Maternity Grant introduction and universalisation were evaluated as distinct natural experimental events, using interrupted time series analysis. The outcome was IMR per 1000 live births. We analysed national data on all infants born in Finland between 1922 and 1975, estimating step and trend changes in the outcome following the point of intervention. Sensitivity analyses included truncating the pre-intervention period and a double break point model, incorporating terms for both introduction and universalisation.

**Results:**

Maternity grant introduction in 1938 was associated with a step-change increase (β=14.59, 95% CI 4.30 to 24.89) in Finnish IMRs. Maternity grant universalisation in 1949 was associated with a step-change decrease (β=−14.35, 95% CI −20.94 to −7.76) in Finnish IMRs. Sensitivity analyses produced corresponding associations.

**Conclusions:**

While we observed changes in IMRs associated with Maternity Grant introduction and universalisation, these changes cannot be disentangled from the impact of the Second World War or other relevant policy developments on infant mortality. Consequently, the relationship between the Finnish Baby Box or comparable contemporary interventions and infant mortality remains unclear.

**WHAT IS ALREADY KNOWN ON THIS TOPIC**

- Interventions modelled on the Finnish Baby Box have seen increasing international uptake, often alongside claims relating to infant mortality and sudden infant death syndrome.

**WHAT THIS STUDY ADDS**

- Despite these claims, our study could not reliably estimate the impact of the Finnish Maternity Grant (which includes the Finnish Baby Box) on infant mortality.

**HOW THIS STUDY MIGHT AFFECT RESEARCH, PRACTICE OR POLICY**

- Our study underscores that the causal relationship between baby boxes and infant mortality remains unclear. However, future research should consider if other health benefits might still be possible.

## Background

The Nordic nations were forerunners in Europe’s 20th century infant mortality decline, despite having comparatively low levels of economic prosperity.^1 2^ At the start of the 20th century, infant mortality rates (IMRs) were higher in Finland than in the other Nordic nations. For example, the mean IMR between 1915 and 1920 was 114 deaths per 1000 live births in Finland, 92 in Denmark, 68 in Iceland and Sweden, and 58 in Norway.^3^ However, by the end of the 20th century, Finland had eventually converged and currently has one of the lowest IMRs in the world (2 deaths per 1000 live births). While a range of medical and social developments likely contributed to 20th century declines in Finnish infant mortality, only the impact of improved urban sanitation has been formally evaluated.^4^

Speculation has recently surrounded the causal role of the Finnish Baby Box in the decline of Finnish IMRs.^5^ Interventions modelled on the Finnish Baby Box have seen increasing international uptake, often aimed at preventing sudden infant death syndrome.^6 7^ Scotland’s Baby Box scheme is currently the only other universally available and non-commercial policy equivalent to the Finnish Baby Box. Introduced in 2017 by the Scottish National Party, it was premised on the purportedly ‘proven’ relationship between the Finnish Baby Box and declining Finnish IMRs.^8^ However, representatives from Finland’s public institutions have questioned this hypothetical relationship and whether it is even empirically verifiable.^9 10^ In this article, we brought natural experimental methods to this debate and evaluated the impact of the Finnish Maternity Grant, the broader policy of which Finnish Baby Box is a component, on Finnish IMRs. Due to a lack of individual-level data, the impact of the Finnish Baby Box could not be evaluated in isolation.

The Maternity Grant was first introduced in Finland in 1938 and later universalised in 1949. Concerns around low birth rates and infant mortality were prominent in Nordic countries at this time.^11–13^ Prior to universalisation in 1949, the grant was means-tested based on family taxable income and only made available to disadvantaged Finnish mothers, who comprised around two-thirds of all new mothers in 1938.^14^ However, uptake levels among the eligible population are not known. From 1942 onwards the grant was received, as it is today, as either the Finnish Baby Box (officially the Finnish Maternity Package) or as a single tax-free cash benefit. In cases of multiple births, both options may be taken. Prior to 1942, care items were offered but were not packaged in a cardboard box.^15^ The nature of the items provided have changed over the years. While the majority of Finnish mothers have opted for the Baby Box since 1974, data for years prior are not available. The Baby Box was not initially intended for infant sleeping, which was instead an innovation on behalf of the parents. Receipt of the Maternity Grant was conditional on obtaining a ‘pregnancy certificate’ from a physician or prenatal clinic, confirming both a pregnancy of >154 days and a health examination undertaken before the fifth month.

Our evaluation of the Maternity Grant aimed to estimate the impact of both introduction and universalisation in 1938 and 1949, respectively, on Finnish IMRs. We hypothesised that, if the Maternity Grant did indeed have a beneficial impact on IMRs, we were most likely to observe this effect following 1938. This was argued from the fact that it impacted a larger proportion of the population than in 1949 and that it targeted disadvantaged mothers, who may be expected to benefit more from receiving the Maternity Grant.

## Methods

### Research design

The introduction and universalisation of the Maternity Grant were evaluated as distinct natural experimental events, using interrupted time series analysis (ITS). ITS is a quasi-experimental method, widely used in the evaluation of healthcare and population-level health interventions.^16–18^ This method forms a counterfactual through an extrapolation of the preintervention outcome trend into the postintervention period. We also used synthetic control (SC) analysis as an alternative approach, which forms a counterfactual with a weighted average of several control populations (see online supplemental appendix 8 for details).^19 20^

10.1136/jech-2022-219488.supp1 Supplementary data

### Data

All data used in this evaluation were sourced from the Human Mortality Database (HMD) (publicly available at: https://www.mortality.org/).^3^ HMD provides high-quality annual birth and death estimates aggregated to a national level. These data are harmonised to produce estimates that are standardised across countries, lending to comparative research.^21^

### Outcome

IMR per 1000 live births was the outcome measure. This was calculated as standard:

$$\frac{Numberofdeathsinfirstyearoflife}{Numberoflivebirths}\times 1000$$

### Main analyses

Two distinct time frames were considered in this evaluation. For Maternity Grant introduction in 1938, a preintroduction period from 1922 to 1937 (16 years) and a postintroduction period from 1938 to 1953 (16 years) were chosen; for Maternity Grant universalisation in 1949, a preuniversalisation period from 1922 to 1948 (27 years) and a postuniversalisation period from 1949 to 1975 (27 years) were chosen. In each case we sought maximise the preintervention data used, while permitting the inclusion of the UK as a control population in the SC analyses (UK data were only available from 1922 onwards).

We used a segmented linear regression to measure the preintervention trend, the immediate step change following the intervention and the change in the postintervention trend compared with the preintervention trend.^22^ All models were checked for autocorrelation and normality (online supplemental appendices 2–6).

All analyses were conducted using R V.4.0.3. The data and code for this analysis are publicly available at: https://github.com/MacCaibe/Maternity-Grant-Evaluation.

### Sensitivity analyses

For both Maternity Grant introduction in 1938 and universalisation in 1949, we truncated the preintervention period to 10 years. This sought to understand whether historic trends were consistent with more recent trends. For the 1949 intervention point specifically, a quadratic term was added to the model retrospectively to capture the curvilinear form of the postintervention outcome trend. Finally, a segmented regression with a double break point incorporating both intervention points was used as an alternative to modelling each intervention point in isolation. As an alternative to ITS analysis, a preliminary SC analysis was performed for both Maternity Grant introduction and universalisation following Abadie and Gardeazabal^23^ and Abadie *et al*.^24^ These analyses included 11 European control populations: Belgium, Denmark, France, Iceland, Italy, Norway, Netherlands, Spain, Sweden, Switzerland and the UK. We chose to prioritise the preintervention fit of models (indicated by the Mean Squared Predictive Error or MSPE) and thus used all preintervention outcomes as sole predictors in all SC models (see online supplemental appendix 8 for details).^25^

## Results

The Finnish IMR was high throughout the first half of the 20th century (online supplemental appendix 1). In 1937, a year before the introduction of the Maternity Grant, the IMR was 68.6 deaths per 1000 live births; in 1948, the year prior to universalisation, it was 51.9 deaths per 1000 live births. There was also considerable variation in early compared with later trends (online supplemental appendix 1).

### Introduction of Maternity Grant in 1938

**Figure 1:**
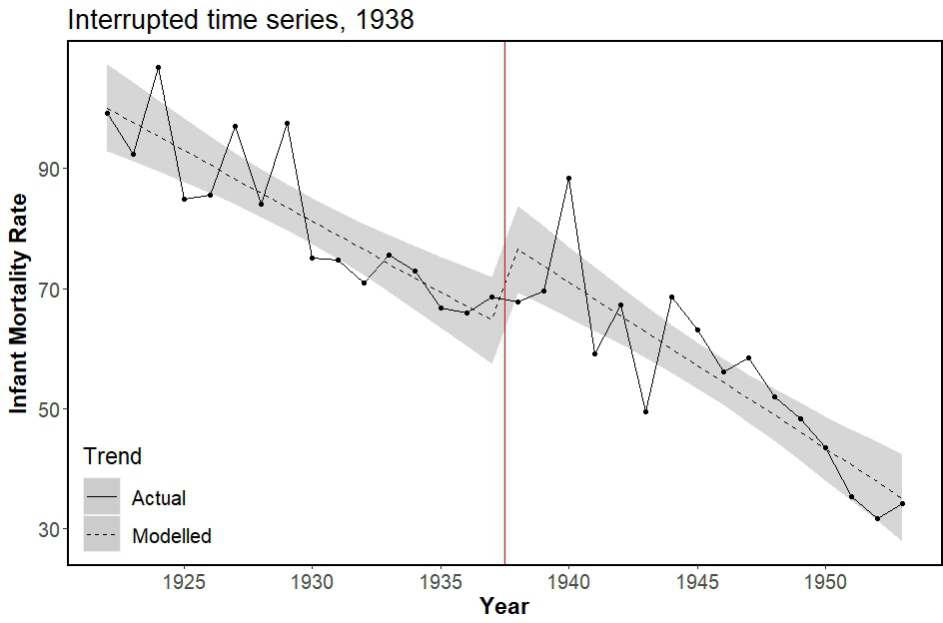
Actual and modelled infant mortality trends (rate per 1000 live births) for main interrupted time series model evaluating Finnish Maternity Grant introduction in 1938 (red vertical line), showing immediate step increase (β=14.59, 95% CI 4.30 to 24.89). Shading represents 95% CI for modelled values.

The main ITS model for Maternity Grant introduction estimated an immediate step increase of 14.59 (95% CI 4.30 to 24.89) deaths per 1000 live births, alongside a marginal and imprecise trend decrease (table 1). Step and trend changes for this model are visualised in figure 1. In 1939, the year after introduction, an absolute difference of 13.8 deaths per 1000 live births was observed (representing a 23% increase on what would have been expected). Both the truncated and double-break point models mirrored this step increase in IMRs, although estimates were smaller (table 1). Residuals for all ITS models evaluating Maternity Grant introduction were normally distributed (online supplemental appendices 2 and 4). The SC model for 1938 exhibited very poor preintervention fit (MSPE=30.19) and could not be interpreted (see online supplemental appendix 8 for details).

**Table 1:** Step change and trend change estimates and 95% CI for all ITS models used in the analysis of Finnish Maternity Grant introduction in 1938 and universalisation in 1949

ITS Model	Introduction 1938		Universalisation 1949	
	Step change (95% CI)	Trend change (95% CI)	Step change (95% CI)	Trend change (95% CI)
Main	14.59 (4.30 to 24.89)	–0.40 (–1.52 to 0.71)	–14.35 (–20.94 to –7.76)	0.40 (–0.02 to 0.82)
Truncation of preintervention trend	10.37 (–3.87 to 24.62)	0.67 (–1.80 to 3.13)	–7.09 (–19.35 to 5.18)	–0.03 (–2.15 to 2.09)
Quadratic term	–	–	–7.88 (–16.33 to 0.56)	–0.94 (–2.16 to 0.29)
Double break point (single model)	10.46 (1.23 to 19.68)	0.44 (–0.82 to 1.70)	–14.77 (–22.68 to –6.87)	0.70 (–0.43 to 1.83)

Autocorrelative function, partial-autocorrelative function and residual plots are available for all models in online supplemental appendices 2–6.

ITS, interrupted time series.

### Universalisation of the Maternity Grant in 1949

**Figure 2:**
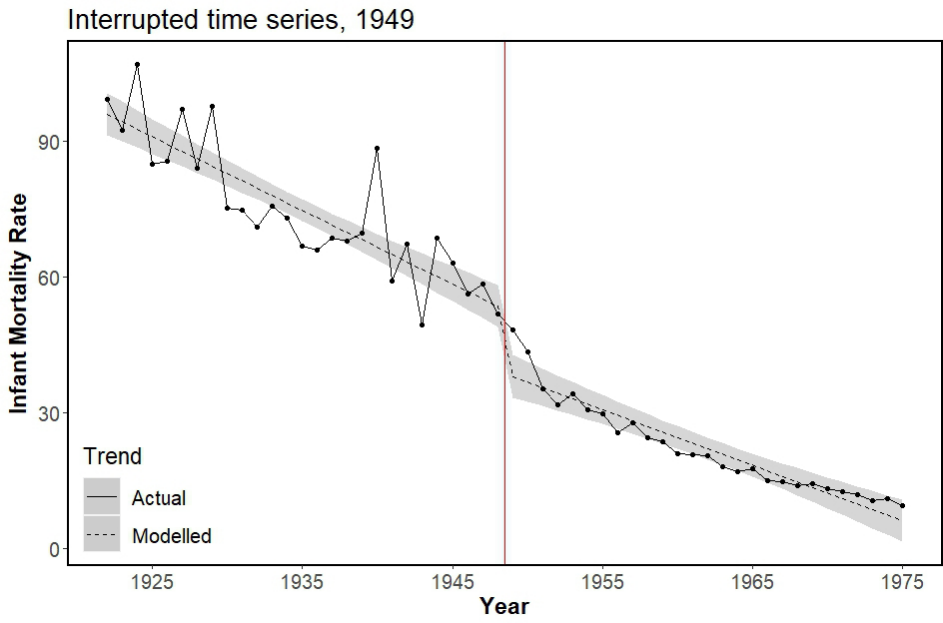
Actual and modelled infant mortality trends (rate per 1000 live births) for main interrupted time series model evaluating Finnish Maternity Grant universalisation in 1949 (red vertical line), showing immediate step decrease (β=−14.35, 95% CI: −20.94 to −7.76). Shading represents 95% CI for modelled values.

The main ITS model for Maternity Grant universalisation in 1949 was associated with an immediate step decrease of −14.35 (95% CI −20.94 to −7.76) deaths per 1000 live births, alongside a marginal trend increase (table 1, figure 2). In 1950, the year following universalisation, the absolute decrease in IMR was 13.54 (representing a 27% decrease on what would have been expected). Model residuals were slightly right skewed (online supplemental appendix 3, plot c). Truncation of the preintervention series estimated a smaller and less precise step decrease (table 1, online supplemental appendix 5, plot c); model residuals were normally distributed for this model, suggesting that variability at the beginning of the study time frame was responsible for the distribution of residuals observed in the main model. Sensitivity models using the quadratic term and the double break point also estimated a step decrease in IMRs following Maternity Grant universalisation. There was no indication of autocorrelation for any ITS models used in this study (online supplemental appendices 2–6, plots a and b). The SC model for Maternity Grant universalisation in 1949 exhibited very poor preintervention fit (MSPE=59.07) and, as before, could not be interpreted (see online supplemental appendix 8 for details).

## Discussion

We evaluated each intervention point as a natural experiment using ITS analysis. Infant mortality was seen to rise following Maternity Grant introduction in 1938. Instead of the Maternity Grant, this may resemble the impact of the Continuation War from 1941 to 1944 and the Lapland War from 1944 to 1945 which likely increased IMRs (eg, through limiting services and resources and through exposure to conflict). Maternity Grant universalisation in 1949 was associated with a decrease in IMRs. However, the influence of other events such as the Child Allowance System—which provided a monthly tax-free cash transfer to mothers from 1948 onwards—and the roll-out of general antenatal care on infant mortality were not accounted for. The latter came into effect in 1945 and likely contributed to falling infant mortality; 86.4% of mothers attended antenatal care in 1945 compared with just 31.3% in 1944.^26^

### Strengths and limitations

We provide the first empirical evaluation of the relationship between the Finnish Baby Box and infant mortality, using high-quality data and robust methods of causal inference. This can be viewed as an example of public health science engaging with a wider policy debate.

Owing to a lack of individual-level data, however, we were unable to isolate the effect of the Finnish Baby Box specifically (as opposed to the wider Maternity Grant). It also meant that, for the evaluation of Maternity Grant introduction, a sizeable proportion of the study population classified as exposed were actually unexposed until universalisation 1949. Conversely, for the evaluation of Maternity Grant universalisation, a sizeable proportion classified as unexposed were actually exposed since introduction 1938. Another major obstacle to causal inference in this study was variability in the outcome trend and the presence of other events relevant to the outcome. For example, it was not possible to account for either the events of the Second World War or other policy developments (eg, the Child Allowance System). Finally, SC models were not interpretable, suggesting a suitable control population (synthetic or otherwise) was not available for this evaluation; this is highlighted by the dissimilarity of the Finnish IMR trend compared with that of the control regions included in the SC models (see online supplemental appendix 7). These limitations prevent any causal interpretation of our findings.

### Policy implications

It cannot be assumed that policies modelled on the Finnish Maternity Grant or Finnish Baby Box reduce infant mortality, as there remains no evidence clearly demonstrating this. The transferability of the Finnish Baby Box to modern contexts should be carefully considered. For example, IMRs were higher in Finland at the time of Maternity Grant introduction than in most low-income and middle-income countries at present.

## Conclusion

The introduction and universalisation of the Maternity Grant were associated with changes in IMRs. However, these associations cannot be interpreted as causal as we did not account for of the impact of the Second World War or other relevant policy developments (eg, the Child Allowance System) on infant mortality. As such, the relationship between the Finnish Baby Box or similar interventions and infant mortality remains unclear.

## Data Availability

Data are available in a public, open access repository. We used national data on annual births and deaths within the first year of life to give infant mortality rates. These data are publicly and readily available from the Human Mortality Database (https://www.mortality.org/). These data follow open data principles.
