# Supplementary material for: Impact of the Finnish Maternity Grant on infant mortality rates in the 20th century: a natural experimental study
Source: J Epidemiol Community Health. 2022 Oct 27;77(1):34–7. doi: 10.1136/jech-2022-219488 (PMC9763162; doi:10.1136/jech-2022-219488)
Supplement: Supplementary data [file jech-2022-219488supp001.pdf]

SUPPLEMENTAL MATERIAL

Appendix 1

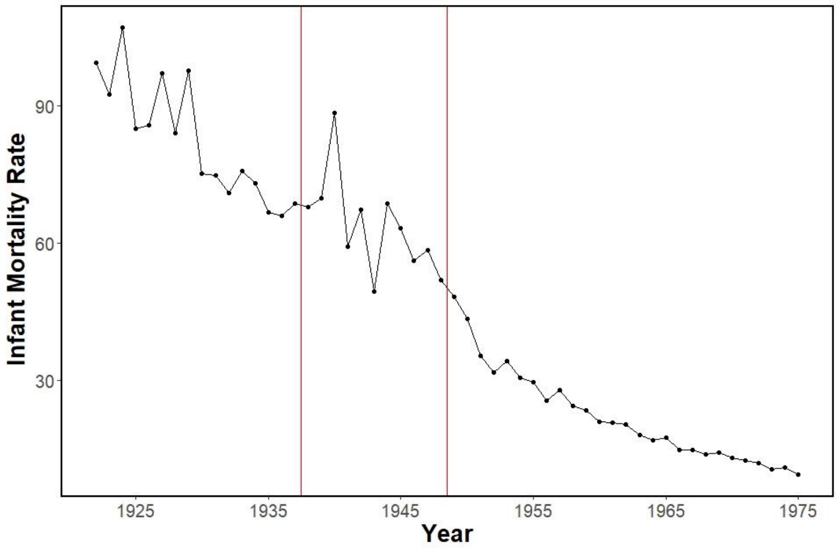

Finnish infant mortality rate per 1000 live births between 1922 and 1975, indicating point of Finnish Maternity Grant introduction in 1938 (left red vertical line) and universalisation in 1949 (right red vertical line).

Appendix 2

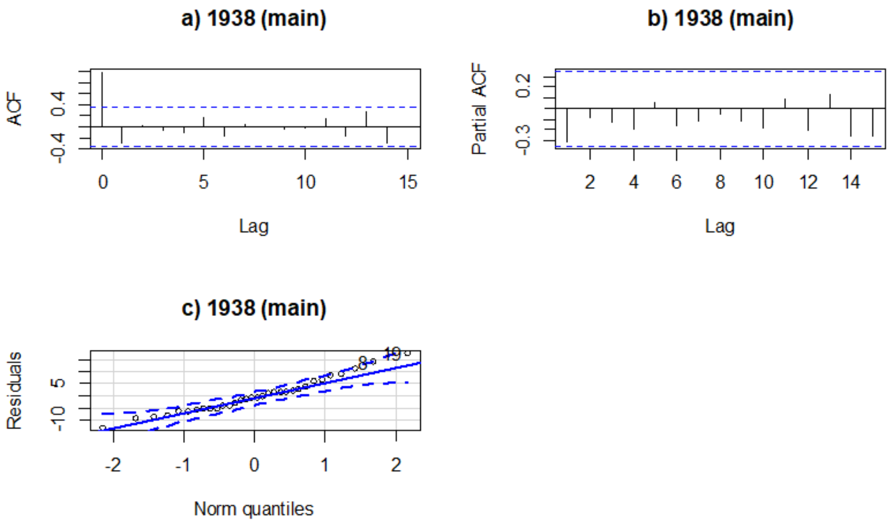

a) autocorrelation function (ACF), b) partial-autocorrelation function (P-ACF), and c) Q-Q plot of residuals for main interrupted time series model evaluating Finnish Maternity Grant introduction in 1938.

Appendix 3

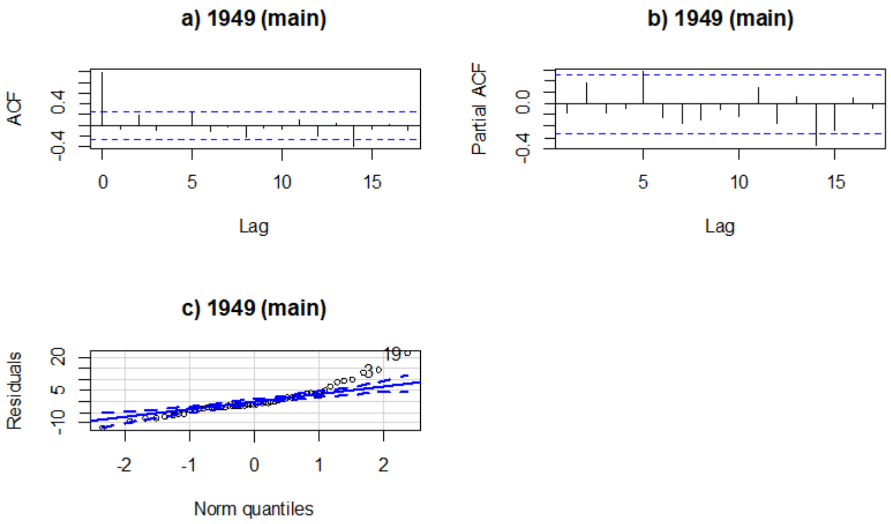

a) autocorrelation function (ACF), b) partial-autocorrelation function (P-ACF), and c) Q-Q plot of residuals for main interrupted time series model evaluating Finnish Maternity Grant universalisation in 1949.

Appendix 4

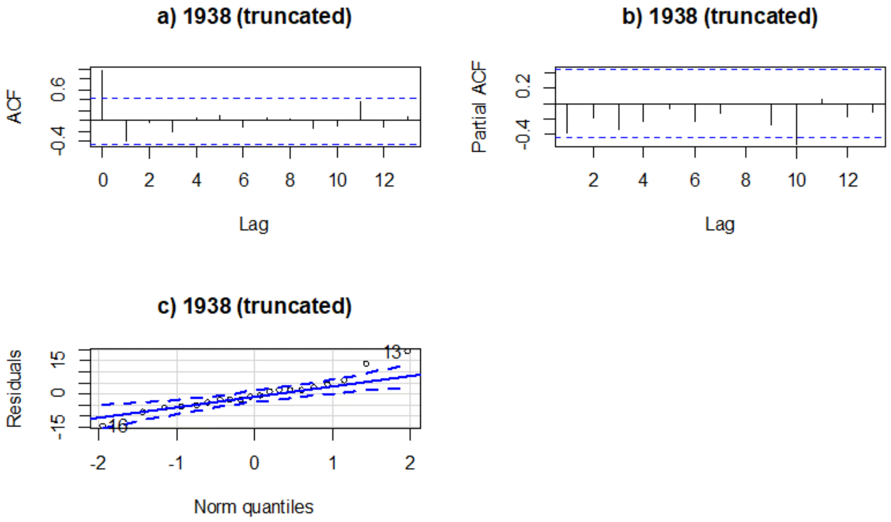

a) autocorrelation function (ACF), b) partial-autocorrelation function (P-ACF), and c) Q-Q plot of residuals for truncated interrupted time series model evaluating Finnish Maternity Grant introduction in 1938.

Appendix 5

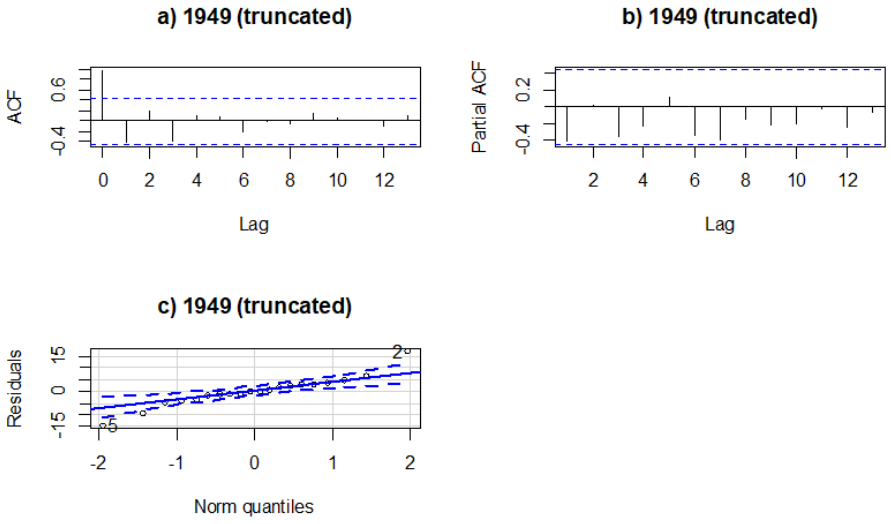

a) autocorrelation function (ACF), b) partial-autocorrelation function (P-ACF), and c) Q-Q plot of residuals for truncated interrupted time series model evaluating Finnish Maternity Grant universalisation in 1949.

Appendix 6

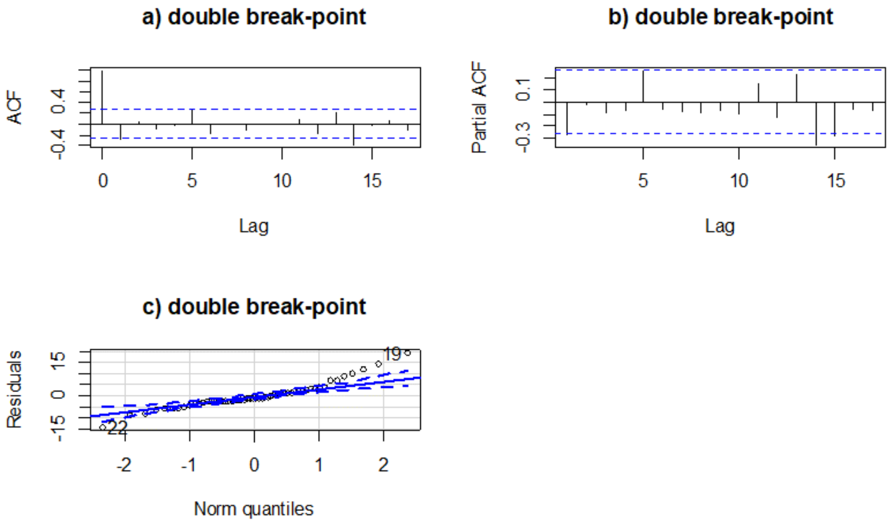

a) autocorrelation function (ACF), b) partial-autocorrelation function (P-ACF), and c) Q-Q plot of residuals for double break-point interrupted time series model evaluating Finnish Maternity Grant introduction in 1938 and universalisation in 1949.

Appendix 7

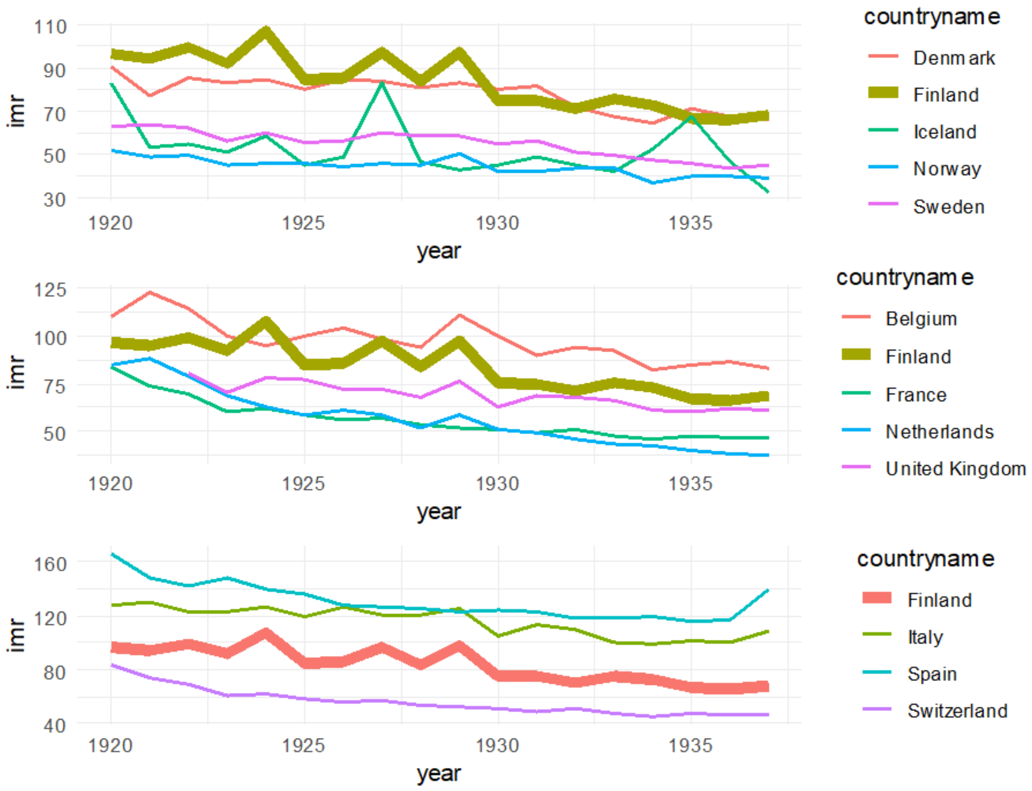

Comparison of infant mortality trends between Finland and potential control countries (grouped by geographical proximity) for the period 1920 to 1938. This visually highlights that none of the potential control countries would be a suitable comparator with Finland.

Appendix 8

SC analyses were conducted following Abadie & Gardeazabal (2007) and Abadie et al. (2011) (references in main text), drawing on eleven European donor populations: Belgium, Denmark, France, Iceland, Italy, Norway, Netherlands, Spain, Sweden, Switzerland, and the UK. All pre-intervention outcomes were used as the sole predictors to optimise pre-intervention fit. Output from these models are presented here as the mean squared predictive error (MSPE) indicating fit, the weights used by the model and a plot of the model trend. Further model diagnostics were not viewed as necessary owing to poor model fit.

Synthetic control for Maternity Grant introduction in 1938

MSPE = 30.19

Weights (%)	Donor country
0.0	Denmark
0.0	Norway
0.0	Sweden
44.5	Netherlands
0.0	France
48.4	Italy
0.0	Spain
0.0	Belgium
7.1	Iceland
0.0	Switzerland
0.0	United Kingdom

Table of weights (%) used in SC by country

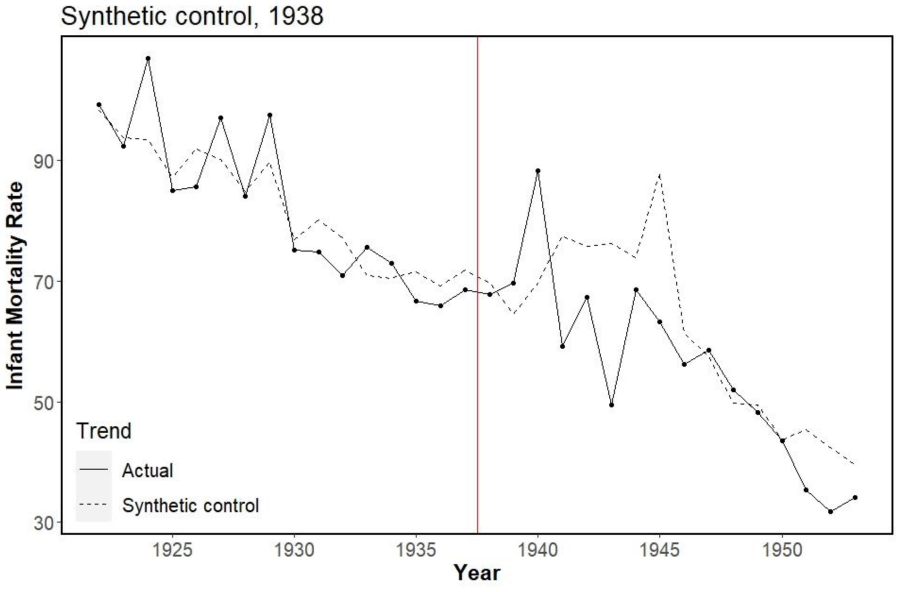

Plot of SC model. Vertical line indicates point of Maternity Grant introduction in 1938.

Synthetic control for Maternity Grant universalisation in 1949

MSPE = 59.07

Weights (%)	Donor country
23.6	Denmark
0.0	Norway
2.2	Sweden
0.0	Netherlands
9.0	France
11.3	Italy
1.6	Spain
34.5	Belgium
5.6	Iceland
9.0	Switzerland
3.2	United Kingdom

Table of weights (%) used in SC by country

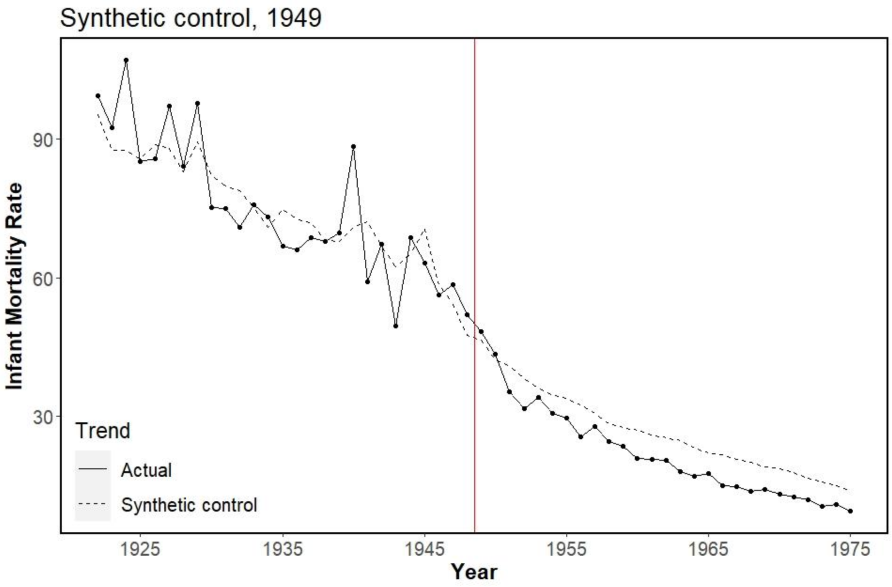

Plot of SC model. Dashed vertical line indicates point of Maternity Grant universalisation in 1949.
